# The Colonic Single Stripe Sign: A Case of Ischemic Colitis

**DOI:** 10.7759/cureus.4622

**Published:** 2019-05-08

**Authors:** Malav P Parikh, Jinendra Satiya, Miguel Berger-Saunderson, Niyati M Gupta, Madhusudhan R Sanaka

**Affiliations:** 1 Gastroenterology and Hepatology, Cleveland Clinic Foundation, Cleveland, USA; 2 Internal Medicine, University of Miami, John F Kennedy Medical Center, Atlantis, USA; 3 Internal Medicine, Cleveland Clinic Foundation, Cleveland, USA

**Keywords:** colonic ischemia, colonoscopy, colitis

## Abstract

Sudden, transient reduction in the blood flow especially in the “watershed” regions of the colon can lead to intestinal ischemia causing a decrease in the delivery of oxygen and nutrients to the intestinal wall. Patients with ischemic colitis often have elevated white blood cell counts, serum lactate, and serum amylase levels. Colonoscopy can confirm the diagnosis of ischemia and the findings include edematous, friable mucosa, erythema with interspersed pale areas, scattered hemorrhagic erosions or linear ulcerations. A single, linear ulcer running along the anti-mesenteric colonic wall, "single-stripe sign"- favors the diagnosis of ischemic colitis. Management of mild and moderate colonic ischemia includes supportive care with bowel rest, gastric suction for associated ileus, fluid-electrolyte balance, and broad-spectrum antibiotics. Patients with severe colonic ischemia may require abdominal exploration and colectomy.

## Introduction

Ischemic colitis is the most common form of vascular injury to the gastrointestinal tract [1]. The most common reason for colonic ischemia is hypoperfusion as compared to thrombotic and embolic events accounting for most vascular insults to the small bowel [2]. Occurrence is typically segmental with left-sided lesions involving the water-shed regions such as the splenic flexure, descending colon, and sigmoid colon. The spectrum of injury ranges from transient colitis, gangrene to fulminant pancolitis. Differential diagnoses for a patient presenting with abdominal pain and bright red bleeding per rectum include inflammatory bowel disease, acute mesenteric ischemia, and infectious colitis. We present the case of a 56-year-old female who presented with bright red bleeding per rectum and was found to have the characteristic single-stripe sign, pathognomonic for ischemic colitis.

## Case presentation

**Figure 1:**
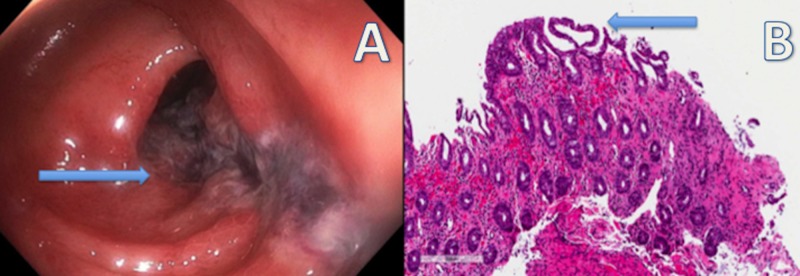
Colonoscopy demonstrating discontinuous areas of non-bleeding ulcerated mucosa with no stigmata of recent bleeding in the sigmoid colon and descending colon; microscopic evaluation revealed characteristic features of surface epithelial injury, crypt epithelial atrophy, crypt loss, lamina propria hemorrhage, and lamina propria hyalinization

A 62-year-old female with a past medical history of tobacco abuse, hypertension, constipation, and chronic headaches managed by ibuprofen, presented to the emergency room with complaints of diffuse abdominal pain and bright red blood per rectum since two days. On physical examination, the patient was afebrile, the abdomen was soft, non-tender, with normal bowel sounds. The rectal exam was unrevealing except for bright red blood on the gloved finger. Laboratory studies showed hemoglobin of 15 g/dl, white blood cell count of 23,700/µl with normal serum lactate, amylase, and lipase levels. Colonoscopy showed discontinuous areas of non-bleeding ulcerated mucosa with no stigmata of recent bleeding in the sigmoid colon and descending colon for a total of 20 cms, consistent with the “single-stripe sign”, characteristic of colonic ischemia (Figure 1A). Further, microscopic evaluation of the biopsied tissue revealed characteristic features of surface epithelial injury, crypt epithelial atrophy, crypt loss, lamina propria hemorrhage, and lamina propria hyalinization consistent with the colonoscopy findings of colonic ischemia (Figure 1B). 

The patient was discharged on a seven-day course of oral ciprofloxacin and metronidazole, managed for constipation with polyethylene glycol, and was advised to quit smoking and to avoid non-steroidal anti-inflammatory drugs.

## Discussion

Sudden, transient reduction in the blood flow especially in the “watershed” regions of the colon can lead to intestinal ischemia causing a decrease in the delivery of oxygen and nutrients to the intestinal wall [3]. The etiology of colonic ischemia can be broadly divided into five categories: 1) non-occlusive ischemia due to hypotension; 2) thromboembolic occlusion of arteries; 3) mesenteric vein thrombosis; 4) mechanical obstruction due to cancer, adhesions or fecal impaction; 4) procedures like aortoiliac reconstruction, abdominal aortic aneurysm repair, renal transplant, and 5) drug-induced ischemia. Common clinical manifestations include abdominal cramping and bright red blood per rectum [4-5]. Transmural infarction can result in gangrenous colitis, peritoneal signs, fever, and ileus. Patients with ischemic colitis often have elevated white blood cell counts, serum lactate, and serum amylase levels. Serum lactate can be used as a predictor of bowel ischemia and is elevated in severe cases of ischemic colitis [6]. Its sensitivity increases in the event of a bowel infarction.

Marston et al. first coined the term ischemic colitis in 1966 to characterize the clinical syndrome associated with vascular compromise to the colon [7]. Zuckerman et al. were the first to report the association of the colonic single-stripe sign with a diagnosis of mild ischemic colitis [8]. They reported the presence of the colonic single-stripe sign greater than 5 cms in length, with 89% of the lesions identified in an isolated segment of the left colon. A preceding ischemic event was noted in 62% of these patients. They believed the linear appearance of the ulcer to be a result of segmental vascular compromise at the mucosal level, occuring due to hypoxemia and small vessel inadequacy. It has been postulated that this edge or demarcation represents involvement of the mesenteric vascular border. Colonoscopy can confirm the diagnosis of ischemic colitis and findings include edematous, friable mucosa, erythema with interspersed pale areas, scattered hemorrhagic erosions or linear ulcerations. A single, linear ulcer running along the anti-mesenteric colonic wall, "single-stripe sign"- favors a diagnosis of mild ischemic colitis.

A plain abdominal radiograph is frequently nonspecific unless there is advanced ischemia, which shows pneumatosis or distension. Computed tomography (CT) of the abdomen with contrast can show edema and thickening of the bowel wall in a segmental pattern, irregular bowel contour, inflamed mesentery with stranding of the fat, or presence of free peritoneal fluid. Management of mild and moderate colonic ischemia includes supportive care with bowel rest, gastric suction for associated ileus, fluid-electrolyte balance and broad-spectrum antibiotics. Patients with severe colonic ischemia require abdominal exploration and colectomy [4-5].

## Conclusions

The increase in longevity of the population has brought about a consequent increase in the incidence of colonic ischemia. Patients commonly present with crampy abdominal pain and rectal bleeding. Colonoscopy is the gold standard for diagnosis. Left-sided lesions in the distribution of the inferior mesenteric artery are more common, while right-sided lesions portend a worse prognosis with a higher risk for surgical intervention. In the majority of patients, treatment is supportive with bowel rest and intravenous fluids.
